# Critical analysis of maternal healthcare delivery systems and mortality patterns: A commentary on research from a tertiary care center in Pakistan

**DOI:** 10.12669/pjms.41.3.11949

**Published:** 2025-03

**Authors:** Abdullah Saad, Dua Fatima Sherwani

**Affiliations:** 1 Abdullah Saad, MBBS 2^nd^ Year Student, Sindh Medical College, Jinnah Sindh Medical University, Karachi 75510, Pakistan; 2 Dua Fatima Sherwani, MBBS 2^nd^ Year Student, Sindh Medical College, Jinnah Sindh Medical University, Karachi 75510, Pakistan

Dear Editor,

We are writing to provide our insights on the article titled “Maternal Mortality: Causes, Trends, and Delays in Care at Tertiary Care Hospital, Pakistan” by Wasim et al., published in the Pakistan Journal of Medical Sciences. This significant study sheds light on maternal mortality statistics, evolving trends over the course of twelve years, and the obstacles women encounter when accessing health services in a prominent hospital in Lahore. While the findings presented are beneficial, we propose that a more profound examination of the root causes and a wider contextual understanding could enhance the depth of this work. Below, we articulate our perspectives in greater detail.

The article discusses a thorough dataset that indicates a maternal mortality ratio (MMR) of 463.92 per 100,000 live births, demonstrating a reduction over time. With the adoption of the “three delays” framework—delays in seeking care, reaching medical facilities, and obtaining adequate treatment—the authors compellingly illustrate the multifaceted nature of maternal fatalities in Pakistan. However, we believe that the research could benefit from a more extensive analysis of the societal and cultural influences that impact health-seeking behaviors. Insights from Kassebaum et al.^1^ and Lawrence et al.^2^ indicate that social norms and perceptions significantly affect women’s health decisions. Incorporating qualitative research that captures community insights could lead to more effective interventions.

Moreover, while postpartum hemorrhage, hypertension, and infections are identified as key contributors to maternal mortality, a more nuanced examination of these issues—particularly regarding differences between urban and rural settings—is essential. Studies indicate that women in rural environments confront distinct challenges due to inadequate access to healthcare facilities.^3^ Recognizing these disparities is critical for developing targeted initiatives that cater to the varied needs of women in these territories.

The increase in maternal mortality recorded in 2022 raises crucial questions regarding its root causes and overall trends. The article’s impact could be amplified by integrating the findings from the Maternal and Perinatal Death Surveillance and Response (MPDSR) system^4^, which may unveil systemic issues that contribute to these deaths. Such additional information could lead to a more comprehensive understanding of the unexpected rise in MMR and could complement findings from other relevant studies.^5^

Lastly, while the authors call for community-focused interventions, providing specific, actionable strategies—like the incorporation of telemedicine services for women in remote areas^6^—would enhance the applicability and relevance of the article’s recommendations.

Additionally, exploring the influence of governmental policies and funding on maternal healthcare could expand the article’s scope. Understanding the role of collaboration between public and private sectors in improving maternal care infrastructure could offer a more holistic view of the issues at hand.

In summary, while Wasim et al.’s research delivers vital insights into maternal mortality in Pakistan, addressing these additional aspects could significantly bolster their conclusions and recommendations. We commend the authors for their valuable contribution and anticipate further research that tackles these urgent public health challenges.
